# Correction: Loss of ErbB3 redirects Integrin β1 from early endosomal recycling to secretion in extracellular vesicles

**DOI:** 10.1083/jcb.20250125508252026c

**Published:** 2026-09-18

**Authors:** Dorival Mendes Rodrigues-Junior, Ana Rosa Sáez-Ibáñez, Julia M. Scheffler, Takeshi Terabayashi, Nina Daubel, Taija Mäkinen, Olof Idevall-Hagren, Aristidis Moustakas, Ingvar Ferby

Volume 225, No. 1 | https://doi.org/10.1083/jcb.202501255 | December 5, 2025

The authors regret that Julia M. Scheffler was inadvertently omitted from the author list in the original version of this article. The corrected author list appears above. The updated author contributions section is as follows, with Julia M. Scheffler’s contributions shown in bold.

Author contributions: Dorival Mendes Rodrigues-Junior: conceptualization, formal analysis, funding acquisition, investigation, methodology, resources, supervision, validation, visualization, and writing—original draft, review, and editing. Ana Rosa Sáez-Ibáñez: conceptualization, data curation, formal analysis, investigation, and visualization. **Julia M. Scheffler: conceptualization, data curation, methodology, and writing—review and editing.** Takeshi Terabayashi: investigation. Nina Daubel: formal analysis and investigation. Taija Mäkinen: resources and writing—review and editing. Olof Idevall-Hagren: formal analysis, funding acquisition, investigation, resources, visualization, and writing—review and editing. Aristidis Moustakas: funding acquisition, investigation, project administration, resources, supervision, and writing—review and editing. Ingvar Ferby: conceptualization, data curation, formal analysis, funding acquisition, investigation, methodology, project administration, resources, supervision, validation, visualization, and writing—original draft, review, and editing.

The error appears in PDFs downloaded on or before August 28, 2026.

